# Manual Dilatation of the Os Uteri as a Means of Inducing Premature Labor

**Published:** 1875-04

**Authors:** A. D. Sinclair

**Affiliations:** M. D., (Harv.), L. M. (Edin.), Fellow of the Obstetrical Societies of Boston and London, etc., etc.


### MANUAL DILATATION OF THE OS UTERI AS A MEANS OF INDUCING PREMATURE LABOR.

BY A. D. SINCLAIR, M. D., (HARV.), L. M. (EDIN.),

*Fellow of the Obstetrical Societies of Boston and London, etc., etc.*

From time to time various means have been resorted to, in order to sufficiently dilate the uterine orifice to admit the hand into the interior of the uterus. Chief among these have been sponge tents and what are known as Keiller's or Barnes' dilators. To the use of these instruments obstetric medicine has been, and will be, greatly indebted, particularly in the operation of inducing premature labor. It is evident, however, that the hand of the obstetrician, as a uterine dilator, may be made a more efficient means, and have claims upon our attention.

1. The hand is always available. 2. It can be easily governed. 3. Wherever a sponge tent or rubber bag can be inserted, the finger may be introduced. 4. The hand and the brain of the operator being in constant sympathy, giving him the precise idea of the condition of the parts, he is enabled to regulate the force necessary to effect dilatation. 5. Artificial dilators frequently require manual aid to keep them in place. 6. As the skilled surgeon, in the process of certain operations, finds greater safety in the use of the fingers than the knife, so may the obstetrician substitute his hand for artificial means as often as practicable.

Delivery was effected by the hand in the following cases: One of convulsions at the seventh month, three of convulsions at the eighth month, two of placenta prævia at the eighth month, one of uterine hemorrhage at the fifth month, one of accidental hemorrhage in the ninth month, and one of persistent vomiting at the seventh month.

Case I. On the 18th of January, 1870, was called to see a primipara, aged twenty-three years, who was seven months advanced in pregnancy, and who had had frequent and severe convulsions for ten hours; the severity of the seizures had been alleviated by chloroform, but the attacks had returned with unabated violence on discontinuing the anæsthetic. She had passed no urine for twenty-four hours, and that obtained from the bladder was very albuminous. The lower extremities were much swollen. The case was regarded as one of extreme danger. The induction of premature labor was advised, as it offered the greatest safety to the patient; the hand was proposed as the instrument to effect delivery. The patient was again chloroformed. The hand being oiled, a finger was passed into the orifice of the vagina, which was small; but after a short time it yielded to gentle pressure sufficiently to admit the whole hand. In the same manner the entrance into the os uteri was effected. Although the opening was at first so small as not to admit the point of the index finger, the exertion of gentle force effected an entrance. Waited until the cervix relaxed, then passed another finger alongside the first, paused until further relaxation ensued, then insinuated a third finger, and so on until the five fingers, wedge-shaped, were all engaged within the cervical cavity.—By gentle force, patiently sustained, the whole hand could now be passed into the cavity of the uterus, but it was allowed to remain in the cervical cavity until complete relaxation had taken place.—This important point should be borne in mind, for on it greatly depends the success of the operation. The child was turned and extracted, and is now living. An hour and a half was occupied in the process, with no evidence that the soft parts had suffered contusion. The only uterine contraction observed was while the hand was in the womb; and deep anæsthesia was required to overcome it. After delivery, the uterus contracted well, and there was no unusual hemorrhage. Convulsions recurred occasionally, for nearly twenty-four hours; they were mitigated by chloroform. The patient made a good recovery, and has since had normal labors.

Case II. On July 26, 1879, saw, in consultation, Mrs. ———, aged twenty-five years. She was eight months advanced in her first pregnancy. She had been healthy till after conception, when she had more or less headache, with gastric disturbance. Her feet began to swell as pregnancy advanced, the œdema being confined to the lower extremities till after the sixth month, when it became general. The urine was scanty and albuminous. Convulsions came on early in the day and continued at short intervals; they were moderated by ether. She was semi-conscious. Immediate delivery by manual dilatation of the os uteri was advised and carried out as in Case I. The cervix was very rigid. The distance between the os externum and the os internum was surprisingly long. An occasional feeble contraction of the uterus was perceived during the delivery, which occupied four hours. The fœtal head presented; version was performed and a still-born child extracted. The uterus contracted normally. After a few hours the patient began to recover consciousness; she had no more convulsions, and appeared to be doing well for seven or eight days; at the end of that time she had profuse hemorrhage from the vagina, which, in the absence of medical aid for some time after its onset, proved fatal.

Case III. In August, 1872, saw Mrs. —, a multipara, who had been suddenly seized with convulsions, an hour or two previous, at the beginning of the eighth month of pregnancy. Ether had already been administered by a physician in attendance. From the albuminous state of the urine and the frequent recurrence of the convulsions, it was agreed that immediate delivery was the safest practice. Under anæsthesia, the hand was used as a dilator of the os uteri, which, after the period of an hour and a quarter, became wide enough to admit the hand into the interior of the womb. The fœtus, whose head presented, was turned and extracted alive. At times, during the process of dilatation, the uterus contracted feebly, and after delivery free hemorrhage came on, which was arrested with difficulty. She made a good recovery, and has since given birth.

CASE IV. On December 25, 1874, was called to advise on the case of Mrs. ——, a young primipara, eight months advanced in pregnancy. Her health had always been excellent until some time after conception. She had had general œdema for several weeks, with headache later on. One day, about noon, she was suddenly seized with convulsions, which recurred at short intervals. She was chloroformed. The urine was almost suppressed; the small quantity taken from the bladder was found to be almost solid with albumen. Three or four hours later, premature delivery was suggested, as the case wore a threatening aspect. This measure, however, was deferred, in the hope that time and other remedies might obviate this last resort, but the convulsions returned with unabated violence and frequency until late in the forenoon of the next day, when delivery was effected by the hand, in a little over an hour.—The child was turned and extracted, still-born. The uterus contracted naturally. There were no further convulsions, and the patient recovered partial consciousness, but soon relapsed into coma and died in nine hours after delivery.

CASE V. On June 29, 1874, was called to see Mrs. ——, aged forty, eight months advanced in her second pregnancy, fourteen years after the birth of her first child. From the seventh to the eighth month, at intervals of a few days, she had had uterine hemorrhage; but during the eight or ten days before she was seen, she had large effusions daily, followed by pallor and exhaustion. For this urgent state of things her physician asked, in succession, two other physicians, who in turn advised the tampon and rest in bed. This course was faithfully pursued without relief. A third physician was then asked in council, who invited the fourth. Ether was administered. After removing an immense plug of rags and clotted blood from an enormously distended vagina, found the uterine orifice firmly closed. By gentle, steady pressure, the finger was readily insinuated into the cavity of the cervix, which in a few minutes relaxed so as to admit another finger, and so on until the whole hand was engaged. Observing the precaution given in Case I., the hand was not passed at once from the cervical into the uterine cavity. Very soon, however, the parts had so relaxed as to warrant the seizure of a fœtal foot. The child was turned and delivery effected in twenty minutes. The placenta was found covering fully three-fourths of the os internum. The uterus contracted well, and as soon as the patient was able to swallow, ergot was given. Mother and child did well.

Case VI. Many years ago, as well as can be recollected (having lost the notes), was called to see a multipara with two physicians who had charge. As there had been much flowing, the apprehension that the case would prove fatal warranted immediate delivery. The cervix uteri was very rigid; nearly two hours was occupied in passing the hand into the uterus, when the child was turned and extracted lifeless. Found that the placenta covered the whole os internum. The patient made a slow but complete recovery.

Case VII. Mrs. ——, aged thirty-four, advanced five months in her third pregnancy, had hemorrhage from the vagina, more or less, for over two months, but for the past fortnight nearly constant and profuse. It was ascertained that several futile attempts had been made to procure abortion, between the second and third months. Taking into consideration the great loss of blood, and the mental condition of the patient consequent on her wrong-doing, was led to the decision, in council with her physician, at once to empty the uterus of its contents. On October 11, 1874, the operation was performed, under ether; the hand was used as the dilator of the cervix, which was exceedingly rigid. Three hours of patient effort was required to effect an entrance, when a fœtus of five months was brought away. The placenta was found everywhere adherent, except at the lower margin, where the finger could be passed beneath it. It was removed by pieces after a good deal of trouble. The patient did well.

Case VIII. On September 4, 1874, was called to see Mrs. ——, a multipara, who had been seized a few hours previous with hemorrhage from the uterus. She was in the ninth month of pregnancy. It was decided, in consultation with her attending physician, that labor should be effected at once, as the hemorrhage continued and labor-pains were wanting. The os uteri was soft and dilatable; the hand was easily passed into the uterus, a fœtal foot seized, the child turned and extracted. The uterus contracted naturally. The process occupied about fifteen minutes. The child appeared still-born, but after a while was resuscitated, and lived about twenty-four hours. Notwithstanding her great loss of blood, the patient made a good recovery.

CASE IX. Mrs. ——, aged thirty-six, a multipara, who had suffered much from vomiting throughout her former pregnancies, had advanced to the seventh month of utero-gestation. The vomiting had been more constant in this pregnancy than any former one, causing great exhaustion. Albumen had not appeared in the urine. Various remedies were tried without relief, and she daily became more prostrate, restless, and wakeful. The pulse, from 140, rose to 160 on the 9th of December, 1870, with a sunken condition of the eyes and ashy hue of the skin. She had the appearance of one in articulo mortis. This truly alarming state of things forced the determination to relieve the uterus of its contents as the only means of safety left to the patient. Without ether, the hand was passed into the uterus and a male fœtus, whose feet presented, was extracted; then a second male child, which presented by the head, was turned and taken away. There was complete absence of uterine contraction during the process of delivery, which took about five minutes. Ergot was given and the uterus contracted well.—One of the twins lived twenty minutes, the other eighteen months. The mother continued to vomit during the next thirty-six hours, and recovery seemed doubtful; but as food was gradually retained in the stomach, after a few weeks she was able to join the family.

All of these cases, except the last, were seen in consultation.

The subject of manual dilatation has been discussed at the meetings of the Obstetrical Society, at various times, for the past four or five years. Several members have adopted the practice under circumstances similar to those detailed in the foregoing cases.—*Boston Medical and Surgical Journal.*

